# Personality and Self-efficacy for Illness Management in Cancer

**DOI:** 10.21203/rs.3.rs-4289523/v1

**Published:** 2024-05-07

**Authors:** Tristen Peyser, Laura M. Perry, Brenna Mossman, Kenneth Xu, Seowoo Kim, James B. Moran, Michael Hoerger

**Affiliations:** Tulane University; Tulane School of Medicine; Tulane University; Tulane University; Tulane University; University of Florida; Tulane University

**Keywords:** Cancer, Motivation, Oncology, Personality, Psycho-oncology

## Abstract

**Objectives:**

Self-efficacy for illness management is increasingly recognized as important for outcomes in cancer. We examined whether The Big Five personality dimensions were associated with self-efficacy for illness management and hypothesized that patients who were less neurotic and more conscientious would have better self-efficacy.

**Methods:**

Adults with cancer completed a cross-sectional survey that included the Mini-International Personality Item Pool (IPIP) and three subscales of the Patient-Reported Outcomes Measurement Information System (PROMIS) Self-Efficacy for Chronic Conditions: managing emotions, managing symptoms, and managing treatment and medication. Linear regressions were used to test the hypotheses, while controlling for covariates.

**Results:**

The personality and PROMIS self-efficacy measures demonstrated good evidence of reliability (median Cronbach’s alpha = .78, range of .69-.92) and validity (intercorrelations). As hypothesized, patients who were less neurotic or more conscientious had higher levels of illness self-efficacy overall and on each of the three subscales (all *p*s < .001). Openness was associated with better self-management of symptoms (*p* = .013) and emotions (*p* = .040). Extraversion was associated with better self-management of emotions (*p* = .024).

**Conclusions:**

Personality plays a vital role in illness self-efficacy for patients with cancer.

**Practice Implications:**

As a part of multidisciplinary care teams, psychosocial experts can use these findings to help patients better manage their illness.

## 1. Introduction

Self-efficacy is associated with better health behaviors and overall quality of life in cancer treatment [1–3]. Self-efficacy can be a valuable predictor of patients’ ability to manage a cancer diagnosis [4] and has been associated with greater physical and psychosocial outcomes [5]. Specifically, patients with greater self-efficacy have an increased ability to manage their cancer-related symptoms, such as fatigue [6] and pain, [7] and manage their emotions through coping and social support [8]. Conversely, patients with lower self-efficacy experience difficulties managing their chronic conditions, treatment, and medication [9]. Therefore, self-efficacy is a critical component of patient illness management that impacts patient well-being and overall quality of life [10]. More fundamental research is needed to understand why people with cancer differ in their capacity for self-efficacy.

Although factors underlying variation in self-efficacy have not been widely studied in cancer populations, a key factor explaining these differences could be personality, one’s relatively enduring pattern of thinking, feeling, and behaving [11]. The Five-Factor Model of personality provides a reasonably comprehensive framework for understanding personality along five core dimensions: neuroticism, conscientiousness, openness, extraversion, and agreeableness [12]. For example, neuroticism refers to feeling chronically sad, worried, angry, or emotionally unstable, and conscientiousness refers to being consistently careful, diligent, thorough, and disciplined. One study found that patients with cancer who had low conscientiousness perceived greater levels of pain severity than those with high conscientiousness and had less reported self-efficacy for pain management [13]. Based on prior research on personality and health, [13–15] we hypothesized that patients who were less neurotic and more conscientious would have overall higher levels of self-efficacy for managing their illness. Moreover, self-efficacy for illness management is a multidimensional construct that involves managing emotions, symptoms, and treatment and medication [16] and this investigation explored how personality was associated with each of these key elements of self-efficacy. Findings may have implications for targeted and tailored interventions to improve illness self-management.

## 2. Material and methods

### 2.1. Participants

The analyses were conducted using baseline cross-sectional data from a randomized controlled trial (Clinicaltrials.gov identifier: NCT04625439). The research was reviewed and approved by Tulane University’s Institutional Review Board (IRB #: 2020-909) in accordance with the Declaration of Helsinki. Participants were recruited online via ResearchMatch.com, the National Institutes of Health (NIH) online participant pool, as well as other health-related and cancer education websites, online support groups, email listservs, and social media. Inclusion criteria included at least 18 years of age and a history of cancer. Informed consent was obtained prior to participation.

### 2.2. Measures

#### 2.2.1. Demographic Characteristics and Disease-Specific Information

Participants reported demographic characteristics relating to their age, gender, race and ethnicity, and education. Participants also reported disease-specific information, including the presence of metastases, cancer type, and years since diagnosis.

#### 2.2.2. Mini-International Personality Item Pool (Mini-IPIP)

The Mini-IPIP is a brief, well-validated 20-item measure of the International Personality Item Pool that assesses the Five Factor Personality model [17, 18]. The Mini-IPIP measures each of the five factors of personality (neuroticism, conscientiousness, openness, extraversion, and agreeableness) using a 4-item subscale [18]. The items are written as statements describing a person’s general tendencies, such as “Get upset easily” (neuroticism) and “Get chores done right away” (conscientiousness), on a scale of 1 (*very inaccurate*) to 5 (*very accurate*) [19]. Total scores were calculated by summing items.

#### 2.2.3. The Patient-Reported Outcomes Measurement Information System (PROMIS) Self-Efficacy for Managing Chronic Conditions

The NIH PROMIS self-efficacy for managing chronic conditions illness management scale assesses an individual’s confidence in managing various aspects of illness, such as symptoms, emotions, and treatments [16, 20]. Subscales for self-efficacy for illness management include managing emotions, managing symptoms, and managing treatments and medications. Custom 4-item short forms using a five-point Likert response scale ranging from 1 (I am not at all confident) to 5 (I am very confident), where participants answered sample items such as “I can find ways to manage stress.” Were used for each domain of self-efficacy for illness management. A total score was summed for each of the domains of self-efficacy for illness management (managing emotions, managing symptoms, and managing treatment and medications). In addition, a self-efficacy for chronic conditions outcome variable was created using a mean composite T-score of the three subscales of self-efficacy for illness management.

### 2.3. Data analyses

Data were analyzed using IBM SPSS Statistics for Windows, version 27 (IBM Corp., Armonk, NY, USA). First, we examined descriptive statistics and correlations to characterize the data. For the personality and PROMIS measures, we also evaluated internal-consistency reliability (i.e., Cronbach’s alpha) and validity, with the personality measures expected to have low intercorrelations with each other (representing distinctness or ‘discriminant validity’) and the self-efficacy measures expected to have high inter-correlations with each other (representing construct similarity, or ‘convergent validity’). Then, for hypothesis testing, we used linear regression analyses. A separate model was used for each dependent variable: self-efficacy for managing emotions, self-efficacy for managing symptoms, self-efficacy for managing treatment and medication, and overall self-efficacy for managing chronic conditions. In each model, the five personality dimensions were predictor variables. In addition, the following covariates were selected based on prior research [21–23] and included in all models: age, gender, education (i.e., presence of bachelor’s degree), presence of comorbidities, years since diagnosis, presence of metastases, and the two most common cancer types in the sample (i.e., breast cancer and genitourinary/gynecologic). The two-tailed alpha level was .05.

## 3. Results

### 3.1. Sample Characteristics

Participants included 372 patients diagnosed with cancer (Table 1). Of these patients, 27.7% were male, the mean age was 58.40 (SD = 13.5), and 94.4% were Caucasian. In addition, patients were primarily college educated (82.5%, n = 307). This sample’s two most common types of cancer were breast cancer (33.1%, n = 123) and genitourinary/gynecologic cancer (29.3%, n = 109). In addition, 81.5% (n = 303) of patients reported no metastases or spread of disease present, and 68.5% (n = 255) of patients reported a cancer diagnosis within the last ten years. Table 1 presents further details of patient demographics.

### 3.2. Bivariate Associations

Table 2 displays the results of the bivariate correlation analysis. The personality and self-efficacy measures had good internal consistency reliability, with an average Cronbach’s alpha of .780. The five personality dimensions had good discriminant validity from each other with a low average inter-correlation (average magnitude *r* = .124), meaning they were measuring distinct constructs. The self-efficacy measures had good convergent validity with an average intercorrelation of *r* = .782, meaning they were assessing similar constructs. Each personality dimension significantly correlated with the total score on self-efficacy for illness management, with the pattern of correlations varying across the three self-efficacy subscales.

### 3.3. Regression Analysis

As hypothesized, linear regression analyses revealed significant relationships between personality and self-efficacy, most notably for neuroticism and conscientiousness. Table 3 shows that neuroticism was a significant predictor for all three self-efficacy subscales and the composite measure: managing emotions (β = −.591, p < .001, 95% CI [−.675, −.504]); managing symptoms (β=−.490, p < .001, 95% CI [−.580, −.398]); managing treatment and medication (β=−.319, p < .001, 95% CI [−.422, − .216]); and self-efficacy for managing chronic conditions composite (β=−.518, p < .001, 95% CI [−.605, −.430]). Conscientiousness was also a significant predictor for all three self-efficacy domains and the composite measure: managing emotions (β = .160, p < .001, 95% CI [.079, .240]); managing symptoms (β = .216 p < .001, 95% CI [.130, .301]); managing treatment and medication (β = .212, p < .001, 95% CI [.115, .309]); and managing chronic conditions composite (β = .223, p < .001, 95% CI [.139, .305]). Openness significantly predicted self-efficacy for managing symptoms (β = .107, p = .013, 95% CI [.022, .192]) and managing emotions (β = .084, p = .040, 95% CI [.003, .163]). Extraversion significantly predicted self-efficacy for managing emotions (β = .091, p = .024, 95% CI [.012, .168]) and managing chronic conditions composite (β = .086, p = .037, 95% CI [.005, .165]). The presence of metastases was the only sociodemographic and disease-specific factor significantly associated with self-efficacy for managing emotions (β = .086, p = 0.027, 95% CI [.010, .162]).

## 4. Discussion and Conclusion

### 4.1. Discussion

Our findings highlight the relationship between personality and self-efficacy for managing illness in the context of cancer. Four personality dimensions were found to predict key elements of self-efficacy for chronic conditions. As hypothesized, patients who were less neurotic or more conscientious had greater overall self-efficacy for managing their illness, including managing emotions, symptoms, and treatments and medications. Additionally, patients who were more extraverted and open had greater self-efficacy, but it was more narrowly confined to specific aspects of managing their illness. Self-efficacy has been assessed in patients with chronic conditions [24–26]. To the best of our knowledge, this is the first study showing that personality is associated with self-efficacy for illness management in cancer. These findings illustrate the importance of personality for understanding variation in self-efficacy for illness management and have implications for how psychosocial experts can improve patient care as a part of multidisciplinary care teams.

First, the findings show that patients with cancer who are less neurotic tend to have higher self-efficacy for managing their illness. These findings expand on previous research demonstrating that personality is associated with engagement in health behaviors among adults with cancer [27, 28]. In particular, lower neuroticism has been associated with better health behaviors (e.g., exercise and diet) and mental health [19, 28, 29]. It is possible that patients who are more neurotic may feel discouraged, hopeless, or too preoccupied with emotions to focus on managing their health more effectively. It was notable that, of all the personality dimensions, it was neuroticism that was most strongly associated with self-efficacy. This finding highlights the critical importance of enduring emotional states in how people manage an illness.

Additionally, conscientious patients had better self-efficacy for managing their illness. This finding builds on prior studies showing that conscientiousness is associated with better health behaviors and mental health [19, 28, 30, 31]. Conscientious individuals tend to be more orderly, diligent, and dutiful. These are important assets across many life domains, and in the context of cancer, amount to better self-management of stress, physical symptoms, and treatments. Patients who struggle more with conscientiousness may benefit from greater external supports in managing an illness.

Furthermore, open and extraverted patients had more self-efficacy, but only in certain domains. Patients who were more open (curious, imaginative, adventurous) had better self-efficacy for self-management of emotions and physical symptoms. It could be that open patients are more disclosing or self-reflective of their emotions and physical illness experience. Further, patients who are more open tend to be more willing to explore various treatment options, which may engender greater confidence in their ability to manage physical symptoms [32, 33]. Extraverted patients had better self-efficacy for self-managing emotions, which could be due to stronger social supports, optimism, or generally better emotional well-being that may accompany extraversion [34]. Given that four personality dimensions were associated with self-efficacy for illness management, these findings highlight the psychological complexity of managing an illness effectively.

#### 4.1.1. Study limitations

This study had several strengths and limitations. Strengths included using a well-validated personality measure arguably underutilized in cancer research, as well as the use of the multifaceted PROMIS self-efficacy measure. The key limitations were that the sample was disproportionately white, female, predominantly had breast, genitourinary, or gynecologic cancers, and was heterogeneous with regard to how long individuals had been living with cancer. Future research should better involve racially and culturally diverse populations and patients with additional cancer diagnoses. Despite these limitations, the findings of our study contribute evidence about personality dimensions that may influence a patient’s illness self-efficacy for illness management.

### 4.2. Conclusion

Personality underlies self-efficacy to manage an illness. Patients with cancer who were less neurotic, more conscientious, more open, or more extraverted had better self-efficacy for managing aspects of their illness. Findings suggest the value of involving psychosocial experts on multidisciplinary care teams to target important personality dimensions, such as neuroticism and conscientiousness when providing care.

### 4.3. Practice Implications

Implications of our study include involving clinicians with personality expertise and integrating brief personality measures in cancer care to better understand how a patient’s personality could inform their illness management. For example, patients with higher neuroticism may benefit from support groups, counseling, and other available psychosocial programs. Patients who are lower in conscientiousness could benefit from notes, reminders, and other infrastructure to facilitate planning, order, and structure. Patients who are less open may need more reassurance to disclose emotional and physical symptoms or try new treatments. Patients who are less extraverted (i.e., introverts) may benefit from problem-solving about how they wish to manage emotions. Multidisciplinary care teams have benefits for cancer management, [35] and the findings highlight some of the specific ways that psychosocial experts can contribute to better patient care. Understanding personality could be a valuable education tool for healthcare providers and, in the case of our results, self-efficacy. Therefore, healthcare providers should understand the role of personality to reduce bias towards patients when not adhering to treatment or specific health behaviors.

## Figures and Tables

**Table 1:** Patient demographic and disease characteristics

Patient characteristics	M (SD) or N (%)

**Age**	58.40 (13.5)
**Sex**	

Male	103 (27.7%)

Female	269 (72.3%)

**Education level**	

Less than high school	5 (1.3%)

High school or GED	14 (3.8%)

Some college but no degree	35 (9.4%)

Certificate or technical degree	11 (3.0%)

College	144 (38.7%)

Graduate	163 (43.8%)

**Race**	

Caucasian	351 (94.4%)

Other	21 (5.6%)

**Cancer type**	

Breast	123 (33.1%)

Genitourinary or Gynecologic	109 (29.3%)

Skin	73 (19.6%)

Colorectal or other Gastrointestinal	38 (10.2%)

Thyroid	27 (7.3%)

Other	19 (5.1%)

**Years since diagnosis**	

Less than 1	40 (10.7%)

1– 4.9	110 (29.6%)

5–9.9	105 (28.2%)

10 or more	117 (31.5%)

**Metastases present**	

Yes	69 (18.5%)

No	303 (81.5%)

Note. N = 372

**Table 2:** Correlation between patient personality, self-efficacy for illness management, and sociodemographic and disease-specific characte

	1	2	3	4	5	6	7	8	9	10	11	12
1. Neuroticism	(739)											
2. Conscientiousness	− .299***	(728)										
3. Openness	− .111	.005	(.717)									
4. Extraversion	− .154*	− .004	.182***	(792)								
5. Agreeableness	− .027	.155**	.139**	.201***	(.689)							
6. Self- efficacy for managing chronic conditions (total score)	− .615***	.387***	.186***	.192***	.111*	(.922)						
6a. Self-efficacy for managing emotions (subscale)	− .654***	.332***	.169**	.200***	.068	.874***	(.823)					
6b. Self-efficacy for managing symptoms (subscale)	− .589***	.374***	.178***	.170**	.088	.913***	.760***	(.830)				
6c. Self-efficacy for managing treatment and medication (subscale)	− .413***	.325***	.150*	.145**	.133**	.871***	.592***	.683***	(783)			
7. Female gender	.027	− .030	− .076	.027	.240***	− .047	− .032	− .035	− .056	-		
8. Age	.320***	.126*	.043	.004	.004	.206***	.164**	.204***	.180***	− .343***	-	
9. Metastases present	.005	− .081	.063	.064	.029	.044	.086	− .007	.039	− .029	− .016	-
10. Education level	− .124*	.050	.245***	.037	.078	.060	.086	.071	.009	− .005	.034	.070
11. Breast cancer	− .135	.040	− .083	.008	.119*	.077	.094	.110*	.010	.409***	− .002	− .115*
12. Genitourinary or Gynecologic	.005	− .035	.073	− .035	− .192***	.001	− .051	.036	.015	− .446***	.339***	− .034
13. Years since diagnosis	− .142	.072	.065	− .002	.031	.163**	.168**	.132*	.135*	− .060	.262***	.059

*Note*: Parenthetical values refer to Cronbach’s alpha internal-consistency reliability

* p < .05

** p < .01

*** p < .001

**Table 3:** Linear regression analyses testing predictors of self-efficacy for illness management

	Self-efficacy in managing chronic conditions		Self-efficacy in managing emotions		Self-efficacy in managing symptoms		Self-efficacy in managing treatment and medication	
*Variable*	β (95% CI)	P	β (95% CI)	P	β (95% CI)	P	β (95% CI)	P
Neuroticism	− .518 (−.605, − .430)	**< .001**	− .591 (−.675, − .504)	**< .001**	− .490 (−.580, − .398)	**< .001**	− .319 (−.422, − .216)	**< .001**
Conscientiousness	.223 (.139, .305)	**< .001**	.160 (.079, .240)	**< .001**	.216 (.130, .301)	**< .001**	.212 (.115, .309)	**< .001**
Openness	.108 (.025, .189)	0.100	.084 (.003, .163)	**0.040**	.107 (.022, .192)	**0.013**	.095 (−.001, .191)	0.054
Extraversion	.086 (.005, .165)	**0.037**	.091 (−.012, .168)	**0.024**	.074 (−.009, .156)	0.082	.065 (−.029, .159)	0.177
Agreeableness	.041 (−.042, .124)	0.330	− .002 (−.083, .079)	0.965	.025 (−.061, .111)	0.564	.080 (−.019, .178)	0.113
*Covariates*								
Age	− .007 (−.106, .091)	0.890	− .062 (−.158, .035)	0.208	.000 (−.103, .103)	0.998	.036 (−.080, .152)	0.544
Gender, female	− .035 (−.130, .060)	0.473	− .048 (−.140, .044)	0.310	− .015 (−.113, .083)	0.760	− .030 (−.141, .082)	0.604
Education	− .055 (−.134, .024)	0.174	.026 (−.103, .051)	0.513	− .039 (−.121, .043)	0.353	− .077 (−.170, .016)	0.108
Comorbidities present	− .047 (−.130, .036)	0.266	− .028 (−.108, .053)	0.505	− .065 (−.151, .021)	0.138	− .033 (−.131, .064)	0.503
Years since diagnosis	.064 (−.016, .143)	0.120	.076 (−.002, .154)	0.057	.035 (−.048, .118)	0.408	.058 (−.036, .152)	0.228
Metastases present	.052 (−.026, .130)	0.192	.086 (.010, .162)	**0.027**	.010 (−.070, .091)	0.801	.043 (−.049, .134)	0.363
Breast Cancer	.022 (−.069, .113)	0.638	.023 (−.066, .112)	0.618	.072 (−.022, .167)	0.135	− .030 (−.137, .078)	0.589
Genitourinary or Gynecologic Cancer	.014 (−.080, .107)	0.774	− .034 (−.125, .057)	0.471	.073 (−.024, .169)	0.143	− .002 (−.111, .108)	0.978

*Note*: The bold values are statistically significant.

## Data Availability

Research data are not shared.
